# “Evaluation of shear bond strength of a composite resin to white mineral trioxide aggregate with three different bonding systems”-An *in vitro* analysis

**DOI:** 10.4317/jced.52727

**Published:** 2016-07-01

**Authors:** Sandhya Yelamali, Anand C. Patil

**Affiliations:** 1 MDS. Conservative Dentistry and Endodontics

## Abstract

**Background:**

Mineral trioxide aggregate (MTA) is a biomaterial that has been investigated for endodontic applications. With the increased use of MTA in pulp capping, pulpotomy, perforation repair, apexification and obturation, the material that would be placed over MTA as a final restoration is an important matter. As composite resins are one of the most widely used final restorative materials, this study was conducted to evaluate the shear bond strength of a composite resin to white mineral trioxide aggregate (WMTA) using three different bonding systems namely the two-step etch and rinse adhesive, the self-etching primer and the All-in-one system.

**Material and Methods:**

Forty five specimens of white MTA (Angelus) were prepared and randomly divided into three groups of 15 specimens each depending on the bonding systems used respectively. In Group A, a Two-step etch and rinse adhesive or ‘total-etch adhesive’, Adper Single Bond 2 (3M/ESPE) and Filtek Z350 (3M ESPE, St Paul, MN) were placed over WMTA. In group B, a Two-step self-etching primer system, Clearfil SE Bond (Kuraray, Medical Inc) and Filtek Z350 were used. In Group C, an All-in-one system, G Bond (GC corporation, Tokyo, Japan) and Filtek Z350 were used. The shear bond strength was measured for all the specimens. The data obtained was subjected to One way Analysis of Variance (ANOVA) and Scheffe’s post hoc test.

**Results:**

The results suggested that the Two-step etch and rinse adhesive when used to bond a composite resin to white MTA gave better bond strength values and the All-in-one exhibited the least bond strength values.

**Conclusions:**

The placement of composite used with a Two-step etch and rinse adhesive over WMTA as a final restoration may be appropriate.

** Key words:**Composite resins, dentin bonding agents, mineral trioxide aggregate, shear bond strength.

## Introduction

Mineral trioxide aggregate (MTA) is a biomaterial that has been investigated for endodontic applications since the early 1990’s (1). It is hard tissue conductive, hard tissue inductive and biocompatible (2,3). Over the years, research on the material has resulted in MTA being applied in various clinical situations like furcation repair, internal resorption treatment, pulpotomy procedures, capping of pulps with reversible pulpitis, apexification and obturation (4-7), in addition to its use as a suitable root-end filling material (8). With the increased use of MTA in pulp capping, pulpotomy, perforation repair, apexification and obturation, the material that would be placed over MTA as a final restoration is an important matter (9). Some of the final restorative materials used in endodontics are Amalgam, Glass Ionomer Cements (resin modified and metal modified Glass Ionomer Cements) and Composite resins (10,11).

Resin composites and glass ionomer cements (GICs) are very popular in restorative dentistry because of their esthetic qualities. Cemal Yesilyurt *et al.* studied the shear bond strength of conventional glass ionomer cements bound to mineral trioxide aggregate allowed to set for 2 different times, 45 minutes and 72 hours. The results of the study showed that the shear bond strength of the conventional GICs to the MTA was similar after 45 minutes and 72 hours and the authors concluded that GICs might be used over MTA after the MTA has set for 45 minutes to allow for single-visit procedures (12).

However, the potential of composite resins which have been widely used as final restorative materials because of their superior physical properties (10,11), to attach to MTA is not well known. Hence the aim of the present study was to evaluate the shear bond strength of a composite resin to white MTA using three different bonding systems namely the two-step etch and rinse adhesive, the self-etching primer and the All-in-one system.

## Material and Methods

The current study was carried out at KLE VK Institute of Dental Sciences, KLE University, Belgaum. The project was cleared by the institutional ethical committee board of KLE University. Forty five specimens of white MTA (Angelus, Brazil) were prepared by using cylindrical acrylic blocks with a central hole measuring 4mm in diameter and 2mm in depth. White MTA was mixed according to the manufacturer’s instructions and filled in each of the prepared acrylic blocks to a depth of 4mm. The filled white MTA was then covered with a wet cotton pellet and a temporary filling material, Cavit – G (3M ESPE, USA) and the specimens were stored at 370C with 100% humidity in an incubator for 24h to encourage setting. After 24h, the temporary material was removed, without rinsing or polishing the surface of the white MTA and the specimens were randomly divided into three groups of 15 specimens each depending on the bonding systems to be used over the MTA surface.

In Group A, the surface of the white MTA was first etched with 37% phosphoric acid (Total-Etch 37% phosphoric acid, Ivoclar Vivadent) and then a two-step etch and rinse adhesive [‘total-etch adhesive’ (Adper Single Bond 2, 3M ESPE, USA)], was applied onto the surface and cured for 20sec. In Group B, a two-step self-etching primer system (Clearfil SE Bond, Kuraray, Japan) and in Group C, an all-in-one system (G Bond, GC Corporation, Japan) was used. All the bonding systems were applied according to the manufacturers’ instructions.

With the help of a cylindrical shaped plastic matrix, a composite resin (Filtek Z350, 3M ESPE, USA) of the dimensions of 2mm diameter and 2mm length was bonded onto the white MTA in all the 3 groups. The polymerized specimens were stored in 100% relative humidity at 370C for 24 hours in an incubator.

The specimens were secured in a holder placed on the platform of the universal testing machine for shear bond strength testing. A knife-edge blade of the dimension 2 mm was used to apply a vertical loading force at a cross-head speed of 1.0 mm/min until the failure of the bond between the composite and the MTA occurred. The peak at which the failure of bond occurred was noted. The shear bond strength in Mega Pascal (MPa) was calculated from the peak bond at failure divided by the specimen surface area and the data obtained was subjected to one way Analysis of Variance (ANOVA) and Scheffe’s post hoc test.

## Results

The mean values and standard deviations of shear bond strengths are given in  Table 1.

**Table 1:**
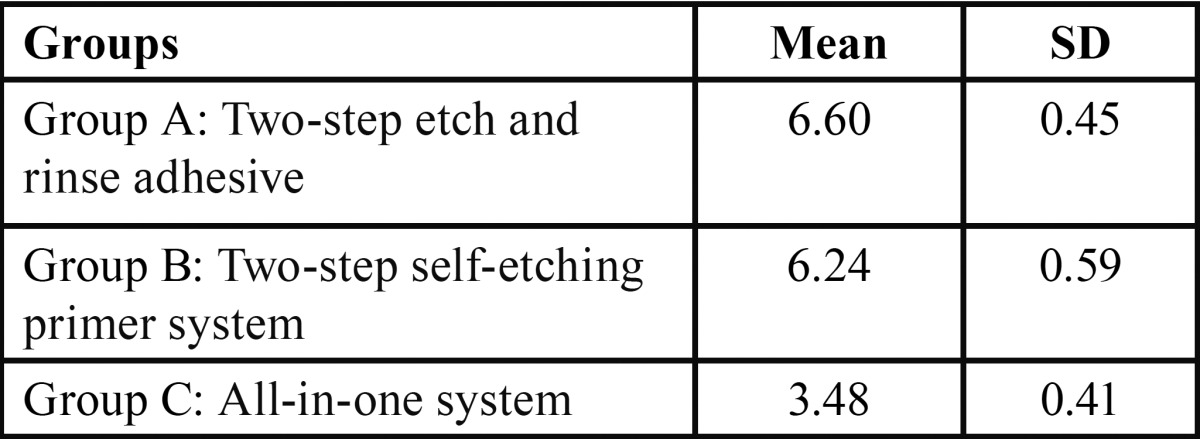
Mean and Standard Deviation (SD) statistics according to study groups (A, B, C) with respect to the shear bond strength values.

The shear bond strengths of the three groups were compared using one way ANOVA ( Table 2). Comparison of the three groups with respect to shear bond strength by using one way ANOVA test revealed that there is statistical significance between all the three groups *p*= 0.0000.

**Table 2:**
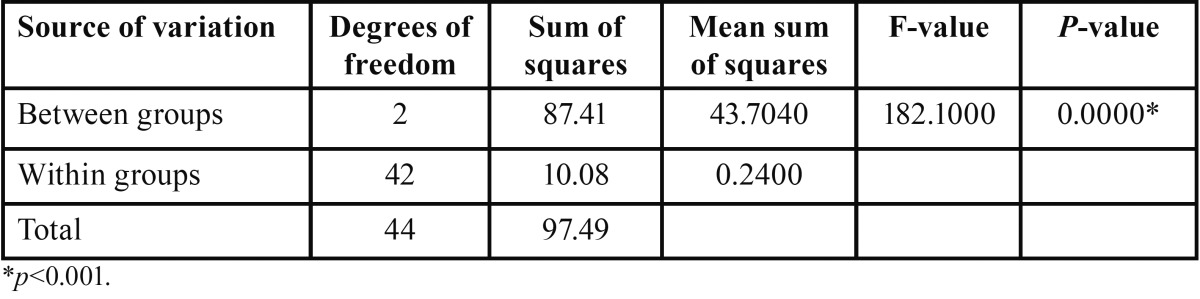
Comparison of three groups (A, B, C) with respect to shear bond strength (in MPa) by one way ANOVA.

To evaluate the difference between pair of groups, the Scheffe’s post hoc test was used ( Table 3). The results showed that the Two-step etch and rinse adhesive (*p*=0.0000) and the Two-step self-etching primer system (*p*=0.0000), had higher bond strength values as compared to the All-in-one system. The values were statistically significant. The Two-step etch and rinse adhesive, as compared to Two-step self-etching primer system had higher bond strength, but was not statistically significant (*p*=0.1447).

**Table 3:**
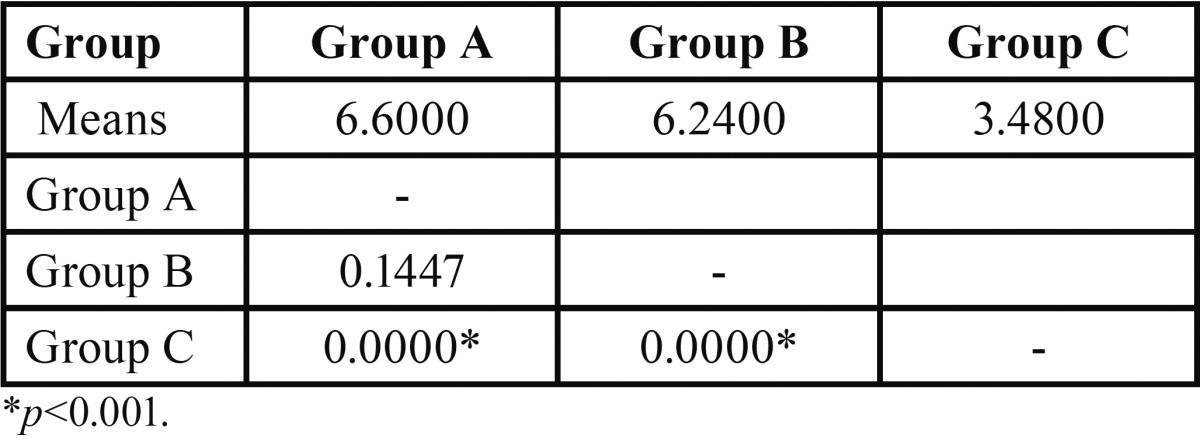
Pair wise comparison of three groups (A, B, C) with respect to shear bond strength (in MPa) by Scheffe’s post hoc test.

## Discussion

Composite resin restorations are required following pulp capping procedures in areas where esthetics is of concern. The bonding between composite resin and the pulp capping biomaterial hence has an important role in quality of fillings and treatment outcomes (9). Sufficient bond strength is required to resist contraction forces to produce gap-free restoration margins. The most common method to evaluate adhesive properties of restorative materials is bond strength assessment (13,14). Hence, the aim of the present study was to evaluate the shear bond strength of a composite resin to white MTA using three different bonding systems namely the two-step etch and rinse adhesive, the self-etching primer and the all-in-one system.

The various commercially available MTA products are; ProRoot MTA which was first introduced, MTA-Angelus, MTA-Angelus Blanco and MTA BIO (8). The use of MTA Angelus in various clinical situations as compared to ProRoot MTA offers certain advantages. The setting time of gray ProRoot MTA was reported to be 2hours and 45min (15), and 2hours and 20 min for white ProRoot MTA (16). Although the manufacturers of MTA-Angelus claim that this material has a setting time of 15 min, there appears to be no independent evidence to confirm this (8) and hence, in the present study MTA-Angelus was left to set for 24 hours as compared to the suggested 48 hours in few of the studies (9,17).

MTA should be kept dry during storage because moist air leads to the phenomenon of air setting, which reduces the strength of the mix. ProRoot products are supplied in single-dose sachets, whereas Angelus products are supplied in double-sealed glass vials. The presentation of ProRoot products as a 1g sachet for single use would result in considerable wastage of material, and the transfer of this material to a sealed container would extend the life of this material and allow more than one treatment to be completed from a single ‘dose’. The Angelus vials are marketed with guidance that 1g may allow up to seven treatments, depending on the volume of material to be used (8).

The other advantage of MTA-Angelus over ProRoot MTA is the cost. MTA-Angelus - gray and white are more economical as compared to ProRoot MTA white (8). Considering these advantages of setting time, cost and storage, white MTA-Angelus was used for the study. 

In the current study the bond strength values between the two-step etch and rinse adhesive and the two-step self-etching primer system was comparable and not statistically significant similar to the findings by Jaberi Ansari Z *et al.* (18), where the shear bond strength of composite to MTA was comparable using etch and rinse and self etch bonding systems.

The results of this study are similar to the findings by Emine Sen Tunc *et al.* (9), where the authors showed total-etch one bottle adhesive system used with a composite resin to bond to MTA gave better bond strength values (13.22 MPa) as compared to the self etch adhesive system used (10.73 MPa). Similarly, a study by Didem Atabek *et al.* (19), concluded that a 2-step total-etch adhesive system exhibited a significantly higher shear bond strength to MTA than the 1-step self-etch and 3-step total-etch adhesive systems.

Lee Seok-Ryun *et al.* (20), studied the effect of acid-etch procedure on the bond between composite resin and mineral trioxide aggregate. The results showed that acid-etch procedure improved the wettability of MTA surface and the bond strength between MTA and composite resin. The authors concluded that acid-etch procedure is essential for a better bond between MTA and composite resin.

The bond strength values for the total-etch system and self-etch primer system obtained in the present study was comparatively lower, that is 6.60MPa and 6.24MPa respectively. This could be due to the MTA Angelus used in the present study as compared to ProRoot MTA used in the study by EmineSen Tunc *et al.* MTA-Angelus consists of 80% Portland cement and 20% bismuth oxide as compared to gray ProRoot MTA which contains 75% Portland cement, 5% calcium and 20% bismuth oxide (21,22). Slightly higher pH and calcium ion release has been seen for MTA-Angelus when compared to ProRoot (22). ProRoot has also been shown to have a more homogeneous composition than gray MTA-Angelus (23). These may have contributed to the low bond strength values seen in the study.

Self-etch systems contain a simultaneously acidic and hydrophilic monomer and do not need to be rinsed away after etching. By decreasing the time and steps required for placement, they have a simplified application method and they are less technique sensitive (24,25). However there is a controversy concerning the efficacy of self-etch systems. Some investigations show that they provide dentin bond strengths comparable with those obtained with the total etch technique (26,27), whereas others have observed significantly lower bond strengths (28,29). The reasons advocated accounting for the suboptimal performance of self-etching primers are: 1. The combination of acidic hydrophilic and hydrophobic monomers into a single step may compromise polymerization of the adhesive, 2. The inherent low strength of the adhesive polymer, 3. The lower degree of polymerization of the resin monomer because of a major solvent/oxygen inhibition effect during light activation of these materials (26). Also, one of the explanations of this low bond strength might be the incompatibility between the adhesive and the restorative material. Hence, the above mentioned reasons could be why Clearfil SE bond, which is a self-etch adhesive, gave lower bond strength values than Adper Single Bond 2, a total-etch adhesive.

Bayrak *et al.* suggested that the nature of the solvent and the filler content of the adhesive might have a greater influence on shear bond strength values than the pH of the adhesive (17). (The pH of G bond is 2.3 and Clearfil SE bond is 1.5) (30). Though G bond and Clearfil SE bond had nearly similar pH values, Clearfil SE bond performed better showing pH alone did not have a greater influence on shear bond strength. Filled, low-viscosity resins are thought to have sufficient strain capacity to relieve stresses between the shrinking resin-based restoration and the rigid substrate (17).

According to Jacobsen (31), bonding systems based on water (ClearfilSE Bond and G Bond) may result in lower bond strength due to incomplete polymerization of the monomers. Furthermore, the water content of WMTA could have interfered with the polymerization of the self-etch adhesives, thereby resulting in reduced WMTA-adhesive bond strength values. Clearfil SE Bond and G Bond contain water, whereas Adper Single Bond 2 is ethanol based. Thus the results were in accordance with Jacobsen (31), where the water based adhesive system showed lower bond strength than the ethanol based adhesive system.

Recently, manufactures have developed self-etching priming resin-based adhesives into a single solution, often referred to as “All-in-one” systems. These adhesives combine the etching, priming and adhesive steps into one process.

Although very simple in technique, studies show that these systems may not perform as well as two-step self-etching priming systems. This is thought to be partially due to water in the adhesive, which is needed to maintain its acidity and also the smear layer being incorporated into the adhesive layer. Simplification of the self-etching priming systems has not led to an improvement in bond strengths (30). In the present study G Bond showed the least bond strength values. This possibly can be overcome by the use of phosphoric acid as recently recommended by the manufacturer to ensure a good enamel bond. However, its effect on dentin bond is not much known, and hence further research is required in this regard.

Within the limitations of the present study, it can be concluded that an etch and rinse (total-etch) adhesive would be the material of choice to attain better bond strength values when bonding composite resin to white MTA.

## Conclusions

Under the conditions of this study, the following conclusions can be drawn

1. Two-step etch and rinse adhesive and the two-step self-etching primer system performed significantly better than All-in-one system in terms of bond strength when used to bond a composite resin to white MTA.

2. Two-step etch and rinse adhesive or ‘total-etch adhesive’ though not statistically significant, gave better bond strength than the two-step self-etching primer system when used as a bonding agent to bond a composite resin to white MTA.
